# Longitudinal effects of atypical antipsychotic drugs on body composition in first-episode and chronic schizophrenia patients

**DOI:** 10.1186/s12888-025-07540-6

**Published:** 2025-12-01

**Authors:** Qi Xiang, Yunhan Ma, Meijun Li, Xiaokun Chai, You Li

**Affiliations:** 1 Department of Nutrition, Tianjin Anding Hospital/Tianjin Mental Health Center, Tianjin, China; 2 Department of Geriatric Psychiatry, Tianjin Anding Hospital/Tianjin Mental Health Center, Tianjin, China

**Keywords:** Schizophrenia, Atypical antipsychotic drugs, Body composition, Central obesity, Metabolic risk

## Abstract

**Objective:**

This study aimed to investigate the effects of atypical antipsychotic drugs (AAPDs) on changes in body composition and fat distribution characteristics in patients with first-episode schizophrenia (FSCZ) and chronic schizophrenia (CSCZ), as well as to predict their temporal trends.

**Methods:**

A total of 70 inpatients (51 FSCZ, 19 CSCZ) admitted to Tianjin Anding Hospital from January 2023 to June 2024 were enrolled. Body composition indicators, including weight (W), Body Mass Index (BMI), waist circumference (WC), hip circumference (HC), waist-to-hip ratio (WHR), abdominal skinfold thickness (ASF), body fat (BF), body fat percentage (BFP), and visceral fat area (VFA) were measured at baseline and every 4 weeks over a 12-week period. A linear mixed-effects model (LMM) was used to analyze dynamic changes, and independent t-tests were used to compare baseline differences.

**Results:**

In FSCZ patients, W, BMI, WC, ASF, BF, and BFP increased significantly over time (*P* < 0.05). The olanzapine group (Group A) showed the steepest slopes for W (1.85 kg/4 weeks), BMI (0.70 kg/m²/4 weeks), and WC (3.07 cm/4 weeks) (*P* < 0.01), indicating central obesity. In CSCZ patients, no significant changes in body composition were observed (*P* ≥ 0.08), but baseline values of W, BMI, and WC were higher than those of FSCZ patients (*P* < 0.05). A LMM predicted that FSCZ patients would reach CSCZ levels in W, BMI, and WC in approximately 64, 42, and 28 weeks, respectively, with other indices reaching similar levels within 22.2–67.7 weeks.

**Conclusion:**

In this preliminary longitudinal study, AAPDs - particularly olanzapine - were associated with rapid and significant increases in central obesity among FSCZ patients. whereas CSCZ patients exhibited metabolic stability over the short observation period. These findings suggest that both disease stage and treatment phase play critical roles in shaping body fat distribution trajectories. The early phase of antipsychotic treatment may represent a vulnerable period for targeted metabolic monitoring and timely intervention to prevent long-term cardiometabolic risks. While these results provide preliminary evidence for stage-specific metabolic patterns, confirmation in larger, randomized, and longer-term studies is required.

## Introduction

Schizophrenia (SZ) is a complex psychiatric disorder with multifactorial etiology, and antipsychotics remain the cornerstone of treatment [1]. Atypical antipsychotic drugs (AAPDs), including olanzapine, risperidone, and blonanserin, are widely used due to their relatively lower incidence of extrapyramidal symptoms. However, they are frequently associated with adverse effects such as weight gain and metabolic syndrome (MetS) [2]. MetS significantly increases the risk of cardiovascular disease, diminishes treatment adherence and quality of life, and poses a major challenge in the management of SZ [3]. Different AAPDs exert varying degrees of metabolic side effects. Olanzapine is known for its high metabolic risk and substantial weight gain; risperidone poses a moderate risk, while blonanserin is associated with relatively mild effects [4, 5]. Most existing studies focus on the general effect of weight gain, while few have explored dynamic changes in fat distribution—especially central obesity—which plays a pivotal role in the development of MetS. Central obesity is closely linked to cardiovascular disease and diabetes and is a critical target for early metabolic management in SZ patients [6]. Patients with first-episode schizophrenia (FSCZ), being newly exposed to AAPDs, may be more sensitive to these metabolic side effects, whereas chronic schizophrenia (CSCZ) patients, due to long-term treatment, typically present with higher baseline body composition and may demonstrate different trajectories. However, longitudinal comparative studies focusing on fat distribution between FSCZ and CSCZ patients remain limited. Quantitative evidence predicting dynamic body composition changes and metabolic outcomes is also lacking [4, 5].

Therefore, this longitudinal study aimed to: (1) characterize body composition changes over time in FSCZ and CSCZ patients receiving AAPD treatment, and (2) estimate the time required for drug-naïve FSCZ patients to reach a body composition profile similar to that of stabilized CSCZ patients. FSCZ was defined as the initial psychotic episode in patients with no prior systematic antipsychotic exposure, whereas CSCZ was defined as illness duration ≥ 5 years with ongoing long-term AAPD therapy (see Methods for details). Particular attention was given to fat distribution, especially central obesity (e.g., WC, VFA) and its cardiovascular relevance, beyond general measures such as weight and BMI. The overarching goal was to generate evidence supporting early metabolic monitoring and intervention in SZ.

## Methods

### Participants

This study was approved by the Ethics Committee of Tianjin Anding Hospital. Patients hospitalized between January 2023 and March 2024 were included. Participants were consecutively recruited from eligible inpatients, with FSCZ recruited from acute admissions and CSCZ from chronic wards to reflect real-world stage differences.

#### Inclusion criteria

(1) Diagnosis of SZ or SZ spectrum disorder based on DSM-5, confirmed by two psychiatrists using SCID-5-CV; (2) Aged 18–70 years, no gender restrictions; (3) FSCZ patients were defined as those with no prior systematic antipsychotic treatment (i.e., no continuous use for > 2 weeks or cumulative use > 4 weeks) and medication-free for at least 2 weeks before enrollment; (4) CSCZ patients were defined as those with illness duration ≥ 5 years and ongoing long-term AAPD treatment (e.g., olanzapine, risperidone, or blonanserin) as part of their standard regimen; (5) Provided written informed consent.

#### Exclusion criteria

(1) Presence of serious neurological, metabolic (e.g., diabetes, hyperlipidemia), hepatic, renal, hematologic, infectious, or malignant diseases; (2) Implanted metal devices interfering with body composition analysis; (3) Inability to cooperate with assessments or discontinuation of medication during treatment; (4) Failure to sign informed consent.

A total of 73 FSCZ patients were recruited, with 51 completing the study (22 dropouts); 57 CSCZ patients were screened, with 19 completing the study (38 excluded due to berberine use).

### Study design

This was a prospective observational study. Patients received standardized inpatient management, including regulated routines and a daily caloric intake of 2000–2500 kcal. FSCZ patients were prescribed olanzapine, risperidone, or blonanserin based on clinical evaluation; CSCZ patients continued their original AAPD regimen. Dosages: olanzapine (5–20 mg/day), risperidone (2–6 mg/day), and blonanserin (8–24 mg/day), adjusted according to efficacy and tolerability. Observation period for all patients was 12 weeks.

### Data collection

Body composition was assessed at baseline and at weeks 4, 8, and 12 following standardized protocols:

- Body weight (W) and height were measured with a calibrated electronic scale, with participants in light clothing and without shoes. Body mass index (BMI) was calculated as weight (kg) divided by height (m²).
- Waist circumference (WC) was measured at the midpoint between the lower rib margin and iliac crest, and hip circumference (HC) at the widest portion of the buttocks, using a non-stretchable tape to the nearest 0.1 cm. Waist-to-hip ratio (WHR) was then derived.
- Abdominal skinfold thickness (ASF) was measured at the right midclavicular line at the umbilical level using a skinfold caliper, with values recorded to the nearest millimeter.
- Body fat (BF), body fat percentage (BFP), and visceral fat area (VFA) were assessed via bioelectrical impedance analysis (InBody S10, Korea).

All measurements were conducted by the same registered dietitian to ensure consistency and minimize inter-observer variability.

### Statistical analysis

All analyses were performed using SPSS version 25.0, with statistical significance defined as *P* < 0.05. All statistical tests were two-tailed. *Given the relatively small sample size*,* effect sizes (Cohen’s d) were calculated to complement P-values and assess the magnitude of group differences.* Longitudinal changes in body composition were analyzed using linear mixed-effects models (LMM), including fixed effects for drug group, time, and their interaction; gender and age were included as covariates to account for baseline differences between groups. Random intercepts were modeled for each subject, with an autoregressive covariance structure of order 1 [AR(1)]. To address the multiple comparisons arising from the nine correlated body composition metrics, the False Discovery Rate (FDR) correction was applied to the p-values for the longitudinal time effects. The validity of the LMM assumptions was further assessed by visual inspection of Q-Q plots and residual-versus-fitted plots for key outcomes. Additionally, residual plots were inspected to validate linearity assumptions. Time to reach CSCZ levels in FSCZ patients was predicted based on the LMM intercepts and slopes.

## Results

### Demographic and baseline characteristics

A total of 51 FSCZ patients (15 males, 36 females) and 19 CSCZ patients (10 males, 9 females) were included. There was no significant difference in gender distribution (χ² = 3.251, *P* = 0.071), but CSCZ patients were notably older than FSCZ patients (*P* < 0.001). At baseline, CSCZ patients had significantly higher values in W, BMI, WC, and other indicators (*P* < 0.05), as shown in Table 1.

**Table 1:** Baseline comparison of body composition metrics between FSCZ and CSCZ patients

Metric	CSCZ (*n* = 19, M ± SD)	FSCZ (*n* = 51, M ± SD)	Mean Difference (95% CI)	Cohen’s d	*p*-value
Age (years)	55.16 ± 5.58	38.18 ± 10.87	16.98 (11.75, 22.21)	1.74	<0.001
W (kg)	71.82 ± 13.39	64.25 ± 11.52	7.57 (4.12, 11.02)	0.62	<0.001
BMI (kg/m^2^)	25.76 ± 4.56	23.59 ± 3.66	2.17 (1.04, 3.30)	0.54	<0.001
WC (cm)	95.10 ± 13.14	84.52 ± 11.19	10.58 (7.20, 13.95)	0.89	<0.001
HC (cm)	97.02 ± 8.81	94.15 ± 7.06	2.87 (0.69, 5.06)	0.37	0.01
WHR	0.927 ± 0.065	0.899 ± 0.073	0.028 (0.008, 0.048)	0.4	0.006
ASF (mm)	43.03 ± 13.29	35.28 ± 11.12	7.75 (4.32, 11.19)	0.65	<0.001
BF (kg)	24.42 ± 9.97	19.83 ± 7.38	4.59 (2.23, 6.96)	0.55	<0.001
VFA (cm^2^)	122.79 ± 53.15	97.70 ± 42.02	25.08 (12.02, 38.15)	0.55	<0.001
BFP (%)	33.15 ± 9.10	30.31 ± 8.36	2.84 (0.39, 5.28)	0.33	0.023

Note: Independent-sample t-tests were used. W = Weight, BMI = Body Mass Index, WC = Waist Circumference, HC = Hip Circumference, WHR = Waist-to-Hip Ratio, ASF = Abdominal Skinfold thickness, BF = Body Fat, BFP = Body Fat Percentage, VFA = Visceral Fat Area

### Baseline comparison among FSCZ treatment groups

FSCZ patients were divided into three groups: Group A (Olanzapine, *n* = 14), Group B (Risperidone, *n* = 24), and Group C (Blonanserin, *n* = 13). No significant differences were observed among the groups in terms of gender or baseline measurements (*P* > 0.05), as presented in Table 2.

**Table 2:** Baseline comparison of age and body composition metrics among FSCZ treatment groups

Metric	Group A (*n* = 14, M ± SD)	Group B (*n* = 24, M ± SD)	Group C (*n* = 13, M ± SD)	F-value	*p*-value
Age (years)	39.43 ± 7.37	38.54 ± 13.15	36.15 ± 9.79	0.322	0.726
W (kg)	61.43 ± 11.87	62.65 ± 11.51	67.58 ± 13.46	0.998	0.376
BMI (kg/m^2^)	22.86 ± 3.65	23.00 ± 3.62	24.19 ± 4.12	0.532	0.591
WC (cm)	81.00 ± 10.94	82.18 ± 10.99	86.81 ± 12.50	0.984	0.381
HC (cm)	94.58 ± 8.81	92.55 ± 7.96	95.54 ± 6.59	0.668	0.518
WHR	0.900 ± 0.078	0.900 ± 0.066	0.885 ± 0.069	0.232	0.794
ASF (mm)	30.08 ± 10.42	31.39 ± 13.15	37.39 ± 9.38	1.544	0.224
BF (kg)	19.13 ± 8.34	18.17 ± 7.29	21.19 ± 7.98	0.638	0.533
VFA (cm^2^)	99.25 ± 47.19	88.45 ± 40.31	100.54 ± 42.34	0.456	0.637
BFP (%)	30.31 ± 9.76	28.48 ± 8.83	30.74 ± 8.27	0.337	0.715

Note: One-way ANOVA was used. W = Weight, BMI = Body Mass Index, WC = Waist Circumference, HC = Hip Circumference, WHR = Waist-to-Hip Ratio, ASF = Abdominal Skinfold thickness, BF = Body Fat, BFP = Body Fat Percentage, VFA = Visceral Fat Area

### Changes in body composition in FSCZ patients

LMM analysis revealed significant time effects for W, BMI, WC, ASF, BF, BFP in FSCZ patients (*p* < 0.05), with significant group-by-time interactions observed for W, BMI, and WC (*p* < 0.01). Group A (olanzapine) showed the steepest slopes: W (1.85 kg/4 weeks, 95% CI: 0.53, 4.18), BMI (0.70 kg/m²/4 weeks, 95% CI: 0.17, 1.57), and WC (3.07 cm/4 weeks, 95% CI: 0.64, 6.78). No significant time effects were found for VFA, HC, or WHR (*p* > 0.05). Sex significantly influenced W and BFP (*p* < 0.01), and age affected WC (*p* < 0.05). Change trajectories and covariate effects are detailed in Table 3. After applying FDR correction to account for multiple testing, only WC (q = 0.032) and ASF (q = 0.001) remained statistically significant.

**Table 3:** Longitudinal changes in body composition metrics in FSCZ patients (*n* = 51)

Outcome	Time Effect (F, *p*)	Group Effect (F, *p*)	Interaction (F, *p*)	Group A Slope (95% CI)	Group B Slope (95% CI)	Group C Slope (95% CI)	Covariate Effects (Sex: F, *p*; Age: F, *p*)
W (kg)	4.926,0.029	1.293,0.283	5.605,0.005	1.85(0.53,4.18)	0.16(-2.02,2.33)	-0.28(-1.24,0.69)	8.404,0.006;2.888,0.095
BMI (kg/m^2^)	5.418,0.022	1.231,0.300	5.538,0.005	0.70(0.17,1.57)	0.08(-0.73,0.89)	-0.10(-0.46,0.26)	0.144,0.706;3.854,0.055
WC (cm)	8.336,0.007	2.343,0.104	5.583,0.008	3.07(0.64,6.78)	0.89(-2.56,4.34)	-0.45(-1.98,1.07)	1.800,0.186;4.496,0.039
HC (cm)	0.058,0.811	1.077,0.347	0.555,0.577	1.29(-1.20,3.78)	0.31(-2.08,2.70)	0.09(-1.11,1.28)	0.001,0.975;0.439,0.511
WHR	0.249,0.621	0.888,0.417	1.259,0.293	0.01(0.00,0.02)	0.00(-0.01,0.01)	0.00(-0.01,0.01)	0.517,0.476;1.771,0.190
ASF (mm)	25.058,0.000	1.363,0.262	0.723,0.493	3.34(-1.18,7.87)	2.67(-1.65,6.99)	1.78(-0.06,3.62)	0.755,0.389;0.087,0.769
BF (kg)	4.580,0.035	1.137,0.328	2.639,0.077	1.04(-0.64,2.71)	0.17(-1.40,1.74)	0.00(-0.69,0.70)	0.973,0.329;1.850,0.180
VFA (cm^2^)	2.528,0.122	0.571,0.568	1.250,0.301	4.48(-6.07,15.04)	0.63(-9.24,10.50)	0.36(-4.01,4.74)	2.471,0.123;3.194,0.080
BFP (%)	4.414,0.047	0.652,0.525	0.805,0.459	0.82(-1.12,2.76)	0.22(-1.60,2.03)	0.28(-0.52,1.08)	11.683,0.001;0.454,0.504

Note: LMM analysis of longitudinal changes, adjusted for sex and age. W = Weight, BMI = Body Mass Index, WC = Waist Circumference, HC = Hip Circumference, WHR = Waist-to-Hip Ratio, ASF = Abdominal Skinfold thickness, BF = Body Fat, BFP = Body Fat Percentage, VFA = Visceral Fat Area

### Changes in body composition in CSCZ patients

No significant time effects were observed for any body composition metrics in CSCZ patients over 12 weeks (*p* ≥ 0.08, range: 0.082–0.955). Sex significantly influenced BF, BFP, and VFA (*p* < 0.05). Change trajectories and covariate effects are detailed in Table 4.

**Table 4:** Longitudinal changes in body composition metrics in CSCZ patients (*n* = 19)

Outcome	Time effect (F, *p*)	Time slope (95% CI)	Covariate effects (Sex: F, *p*; Age: F, *p*)
W (kg)	0.79,0.377	0.38(-0.48,1.24)	0.12,0.739;0.49,0.491
BMI (kg/m^2^)	0.42,0.519	0.10(-0.21,0.42)	1.28,0.274;0.10,0.759
WC (cm)	0.73,0.397	0.45(-0.60,1.50)	0.45,0.511;0.24,0.630
HC (cm)	0.29,0.592	0.12(-0.33,0.58)	0.13,0.727;0.29,0.595
WHR	0.08,0.776	0.00(-0.01,0.01)	0.90,0.356;0.26,0.620
ASF (mm)	3.41,0.082	1.97(-0.28,4.22)	1.17,0.295;1.92,0.184
BF (kg)	0.10,0.752	0.12(-0.63,0.87)	4.98,0.040;0.00,0.980
VFA (cm^2^)	0.00,0.955	-0.11(-3.91,3.70)	7.67,0.013;0.04,0.851
BFP (%)	0.19,0.668	-0.12(-0.69,0.45)	15.53,0.001;0.31,0.582

Note: LMM analysis of longitudinal changes, adjusted for sex and age. W = Weight, BMI = Body Mass Index, WC = Waist Circumference, HC = Hip Circumference, WHR = Waist-to-Hip Ratio, ASF = Abdominal Skinfold thickness, BF = Body Fat, BFP = Body Fat Percentage, VFA = Visceral Fat Area

### Time to reach CSCZ levels in FSCZ patients

To predict the time for FSCZ patients’ body composition to reach CSCZ levels, LMM were employed (Table 5). Linearity assumptions were tested by including quadratic time terms (Time²). Table 6 shows that, except for ASF (*p* = 0.029), Time² and Group × Time² interaction effects were non-significant (*p* > 0.05), supporting linearity. Based on LMM, FSCZ patients’ W, BMI, WC, HC, WHR, BF, FA, and BFP were predicted to reach CSCZ levels in 22.2–67.7 weeks as shown in Table 5. ASF was not predicted due to its non-linear trajectory.

**Table 5:** Predicted time for FSCZ patients to reach CSCZ body composition levels (LMM linear Model)

Metric	CSCZ intercept (M ± SE)	FSCZ intercept (M ± SE)	CSCZ slope (95% CI)	FSCZ slope (95% CI)	Predicted time (weeks)
W (kg)	58.13 ± 8.33	55.01 ± 4.11	0.316[-0.421,1.054]	0.511[-0.732,1.122]	64
BMI (kg/m^2^)	21.76 ± 2.78	20.55 ± 1.37	0.087[-0.187,0.360]	0.203[-0.228,0.461]	41.7
WC (cm)	83.74 ± 8.31	73.25 ± 4.13	-0.402[-1.348,0.545]	1.088[0.288,2.691]	28
HC (cm)	95.69 ± 5.47	91.78 ± 2.67	-0.515[-0.947,-0.083]	0.188[0.112,1.293]	22.2
WHR	0.89 ± 0.05	0.87 ± 0.02	0.001[-0.006,0.007]	0.002[-0.007,0.010]	55.1
ASF (mm)	41.64 ± 7.77	32.08 ± 4.26	1.828[0.255,3.401]	2.624[-1.325,2.915]	—
BF (kg)	20.63 ± 5.64	16.26 ± 2.80	0.110[-0.477,0.696]	0.369[-0.479,0.998]	67.7
VFA (cm^2^)	95.42 ± 30.19	71.67 ± 14.99	-0.157[-3.405,3.091]	1.780[-2.312,5.871]	53.2
BFP (%)	34.95 ± 5.35	29.51 ± 2.66	-0.114[-0.716,0.489]	0.427[-0.213,1.294]	40.3

**Table 6:** LMM tests of Time² and group × Time² effects for FSCZ and CSCZ patients

Metric	Time² Estimate ± SE	Time² *p*-value	Group × Time² Estimate ± SE	Group × Time² *p*-value
W (kg)	-0.454035 ± 0.231814	0.094	0.380444 ± 0.313093	0.226
BMI (kg/m^2^)	-0.155082 ± 0.086685	0.116	0.124876 ± 0.117326	0.289
WC (cm)	0.079030 ± 0.330201	0.518	-0.445417 ± 0.443467	0.317
HC (cm)	-0.505175 ± 0.245418	0.255	0.363827 ± 0.330077	0.159
WHR	-0.006054 ± 0.003110	0.054	0.004497 ± 0.004181	0.115
ASF (mm)	-2.464962 ± 0.824789	0.029	2.557105 ± 1.078098	0.019
BF (kg)	-0.273050 ± 0.186907	0.561	0.398679 ± 0.252873	0.117
VFA (cm^2^)	-0.725516 ± 1.044943	0.956	1.528921 ± 1.413133	0.281
BFP (%)	-0.087588 ± 0.193711	0.753	0.257755 ± 0.261308	0.326

Note: LMM predictions adjusted for sex and age. Intercepts are reported as mean ± SE; slopes with 95% CI. Predicted time calculated as [(CSCZ intercept – FSCZ intercept)/(FSCZ slope – CSCZ slope)] × 4. ASF was not predicted due to significant Time² effect (*p* < 0.05). W = Weight, BMI = Body Mass Index, WC = Waist Circumference, HC = Hip Circumference, WHR = Waist-to-Hip Ratio, ASF = Abdominal Skinfold thickness, BF = Body Fat, BFP = Body Fat Percentage, VFA = Visceral Fat Area

## Discussion

In summary, this longitudinal study describes distinct patterns in body composition changes between FSCZ and CSCZ patients under AAPD treatment. FSCZ patients exhibited significant increases in key metrics such as weight, BMI, and waist circumference, indicative of central obesity, particularly in the olanzapine group. In contrast, CSCZ patients maintained metabolic stability despite higher baseline values. Model projections suggest that FSCZ patients could approach CSCZ levels within 0.5–1.5 years. Notably, following FDR correction, significance persisted only for WC and ASF, consistent with an early redistribution toward central adiposity rather than broad increases in overall adiposity.

## Central obesity in FSCZ patients

This study found significant increases in W, BMI, WC, ASF, BF, and BFP in FSCZ patients over 12 weeks (*p* < 0.05; Table 3). Significant group-by-time interactions for W, BMI, and WC were observed in the olanzapine group (Group A), with slopes of 1.85 kg/4 weeks (*p* = 0.005), 0.70 kg/m²/4 weeks (*p* = 0.005), and 3.07 cm/4 weeks (*p* = 0.008), respectively, indicating AAPD-induced central obesity, particularly with olanzapine. After multiple testing correction, only waist circumference and abdominal skinfold thickness remained statistically significant (q < 0.05), suggesting that early metabolic changes were concentrated in central adiposity rather than in overall body weight or BMI. While increases in body weight and BMI are important markers of general adiposity, the pronounced increase in waist circumference (WC) observed particularly in the olanzapine group is highly clinically relevant. WC is a simple yet effective surrogate marker for visceral adiposity, which is a core component of metabolic syndrome and is independently associated with an increased risk of cardiovascular disease and type 2 diabetes [6]. Our data, showing a steep increase in WC (3.07 cm per 4 weeks) in drug-naïve FSCZ patients starting olanzapine, suggest a rapid onset of central fat accumulation pattern. This shift towards central obesity may partly explain the significantly elevated metabolic risk observed in patients on certain AAPDs, even before substantial overall weight gain occurs. Therefore, monitoring WC, in addition to weight, provides a more targeted assessment of cardiometabolic risk early in the treatment course.

To further validate the robustness of these findings, we applied the FDR correction to address multiple comparisons across the nine body composition metrics. After this rigorous adjustment, the rapid increases in WC (q = 0.032) and ASF (q = 0.009) remained statistically significant, whereas the changes in general adiposity metrics such as body weight and BMI did not. This statistical pattern suggests that a key early metabolic effect of AAPD initiation in drug-naïve FSCZ patients is a rapid shift toward a central fat distribution pattern, rather than generalized weight gain alone. This specificity greatly enhances the clinical relevance of our findings, guiding clinicians to focus monitoring specifically on waist circumference as a key indicator of metabolic risk.

The validity of these conclusions is further supported by diagnostic checks of the linear mixed-effects models. Visual inspection of Q-Q plots for primary outcomes, including WC and ASF, indicated no severe deviations from normality, and their residual-versus-fitted plots showed a random, homoscedastic pattern, supporting the model’s assumptions. A notable exception was the waist-to-hip ratio (WHR), which exhibited a patterned distribution—a known phenomenon for ratio variables with limited decimal precision that does not indicate a model violation. Collectively, the consistency of the effects after FDR correction and the supportive model diagnostics support confidence in the rapid onset of central obesity in first-episode patients.

Our findings on fat distribution align with and extend the well-established literature on AAPD-induced metabolic adverse effects [3, 4, 7]. While prior studies, such as those by Correll et al. [4] and Piras et al. [5], have robustly documented the overall weight-gain potential of olanzapine and risperidone, our study provides a more nuanced longitudinal analysis by specifically highlighting rapid central obesity development in FSCZ patients. This provides an incremental contribution to the current literature, as central obesity is a stronger predictor of cardiovascular risk than overall weight gain [6]. Indeed, previous studies have reported significant weight gain with olanzapine [7, 8]. Our results not only confirm this but further elucidate the specific pattern of abdominal fat accumulation, which may relate to AAPD-associated alterations in neurotrophic signaling (e.g., BDNF) and downstream metabolic–inflammatory pathways reported in prior work; however, we did not assess these biomarkers in the present study, and this mechanistic interpretation should be considered hypothesis-generating [9].

Consistent with the known metabolic risk profile of these medications, risperidone (Group B) and blonanserin (Group C) showed smaller, non-significant slopes (*p* >0.05; Table 3), supporting olanzapine’s higher metabolic risk profile [10, 11]. Compared to FSCZ patients, CSCZ patients showed no significant changes in central obesity metrics (*p* ≥ 0.08; Table 4), despite higher baseline values (e.g., WC: 95.10 cm vs. 84.52 cm, *p* < 0.001; Table 1). This difference highlights similarities in fat distribution patterns influenced by AAPD but underscores the role of disease chronicity, where long-term exposure in CSCZ may lead to a plateau in metabolic alterations, unlike the rapid onset in FSCZ.

In summary, unlike prior studies that focused solely on weight gain in mixed cohorts [4, 5, 7, 8], our study differentiates FSCZ and CSCZ, revealing stage-specific fat distribution; this difference may arise from our longitudinal design and focus on inpatient settings, where diet is controlled, allowing clearer observation of early metabolic effects in FSCZ.

## Baseline differences and metabolic stability between FSCZ and CSCZ patients

At baseline, CSCZ patients had significantly higher values in W, BMI, WC, and other metrics compared to FSCZ patients (*p* < 0.05; Table 1), reflecting the impact of chronic disease and long-term AAPD exposure. Over the 12-week period, CSCZ patients showed no significant changes in body composition (*p* ≥ 0.08; Table 4). This may reflect metabolic adaptation or a “ceiling effect” from prolonged AAPD exposure, where weight changes stabilize [12, 13]. Long-term AAPD treatment may induce leptin resistance via elevated leptin levels, initially promoting hyperphagia but later stabilizing energy intake and expenditure due to the hypothalamic signaling adaptations [14, 15]. These findings align with the non-significant time effects observed in CSCZ patients. Compared to meta-analyses showing persistent weight gain in chronic patients [12, 13], our findings of stability in CSCZ may be due to our shorter 12-week follow-up and standardized caloric intake, suggesting adaptation occurs earlier in controlled environments.

## Sex and age as potential modifiers

In FSCZ patients, females exhibited significantly higher BFP than males (*p* = 0.001; Table 3). In CSCZ patients, females had higher BF, FA, and BFP (*p* < 0.05; Table 4), suggesting greater metabolic risk in females, possibly due to estrogen levels or sex-specific fat distribution patterns [16]. Age significantly influenced WC changes in FSCZ patients (*p* = 0.039), with near-significant effects on BMI (*p* = 0.055) and FA (*p* = 0.080), indicating older FSCZ patients are more susceptible to WC and FA increases, consistent with the positive correlation between age and MetS risk [17, 18].

In CSCZ patients, age had no significant effect (*p* >0.05; Table 4), possibly due to metabolic stabilization from prolonged AAPD exposure. These covariate effects highlight the role of individual differences in AAPD-induced metabolic changes.These sex differences may be attributed to hormonal factors, such as estrogen levels promoting greater fat accumulation in females [16]. Similarly, age-related susceptibility in FSCZ patients could stem from declining metabolic rates and increased inflammation with aging, amplifying AAPD-induced changes [17, 18]. The lack of age effects may reflect adaptation over time, where chronic AAPD exposure alters hormonal responses. These findings extend previous work on sex/age in MetS [16–18], where our covariate analysis highlighted interactions with disease stage, possibly because chronic AAPD exposure alters hormonal responses over time.

## Predicted trajectories for FSCZ patients reaching CSCZ levels

LMM predictions indicated that FSCZ patients’ body composition metrics may reach CSCZ levels within approximately 1 year (22.2–67.7 weeks; Table 5). This aligns with evidence that AAPD-induced weight gain is most rapid in the first few months, slowing thereafter, but may persist for 1–4 years [19]. The non-linear trajectory for ASF may reflect variability in fat deposition or measurement sensitivity, warranting longer-term follow-up studies.

## Clinical implications and study innovations

Previous studies have consistently shown that AAPDs, particularly olanzapine, induce significant weight gain, increased body fat (including visceral fat accumulation), and related metabolic changes such as elevated leptin and insulin levels in SZ patients, with first-episode individuals being especially vulnerable due to drug-naivety [20–23]. However, much of this research has focused on overall weight gain or short-term metabolic syndrome risks, with limited longitudinal comparisons of fat distribution between first-episode and chronic stages or predictions of temporal trends [22–25].

This study’s innovation lies in quantifying central obesity patterns in FSCZ patients and contrasting them with CSCZ patients’ metabolic stability, offering new insights into AAPD-mediated metabolic risk management. WC serves as a practical screening tool for early intervention, particularly in olanzapine-treated FSCZ patients. In contrast, CSCZ patients’ high baseline metrics suggest earlier metabolic interventions are needed. While previous studies focused on overall weight gain, this study elucidates fat distribution dynamics, filling a critical research gap. Our innovation in predicting temporal trajectories of body composition using LMM fills a critical gap in longitudinal quantitative evidence [22–25], with differences likely attributable to our use of dynamic LMM modeling versus static measures in prior studies. These findings may guide early metabolic screening strategies in clinical psychiatry.

## Limitations and future directions

This study has several limitations: (1) the sample size is relatively small, particularly for the CSCZ group (*n* = 19) and the FSCZ subgroups (olanzapine *n* = 14, risperidone *n* = 24, blonanserin *n* = 13), which limits the statistical power of the analyses, increases the risk of Type II errors, and reduces the precision of our estimates; furthermore, the significant baseline differences (e.g., in age) between FSCZ and CSCZ groups introduce inherent sample heterogeneity which, despite statistical adjustment, may leave residual confounding bias; (2) the observational design involved non-randomized allocation of AAPDs to FSCZ patients based on clinical judgment, which may introduce selection bias; (3) the absence of data on metabolic parameters such as glucose, insulin, or lipid profiles prevents direct correlation with the observed body composition changes; and (4) the short 12-week observation period may underestimate long-term changes in CSCZ patients, and there was no adjustment for drug dosage or disease severity; (5) Antipsychotic dose (transformed to chlorpromazine equivalents, CPZE) was not included as a covariate in the LMM analyses due to unavailable detailed dose records, as doses varied during the observation period for some patients, and historical dose data for CSCZ patients were difficult to retrieve; this may introduce confounding bias. Future studies should expand sample sizes to enhance statistical power for subgroup analyses, particularly for CSCZ patients and FSCZ treatment groups, incorporate randomized allocation of AAPDs to minimize selection bias, include metabolic parameters such as glucose, insulin, and lipid profiles to explore their correlation with body composition changes, extend follow-up periods, and adjust for confounders such as drug dosage (transformed to CPZE) and disease severity to validate metabolic adaptation in CSCZ patients and elucidate fat distribution mechanisms. In addition, given that dietary intake and physical activity were not quantitatively controlled beyond standardized meal plans, residual confounding from lifestyle factors cannot be ruled out. Moreover, the observational design precludes causal inference regarding the relationship between AAPD exposure and body composition trajectories. Future large-scale, randomized longitudinal studies are therefore warranted to validate these findings.

## Data Availability

The datasets used and/or analyzed during the current study are not publicly available due to privacy and ethical restrictions but are available from the corresponding author on reasonable request, subject to approval by the Ethics Committee of Tianjin Anding Hospital.
